# An Appeal for £200,000

**Published:** 1916-03-04

**Authors:** L. E. Beaufort, Adeline M. Bedford, S. F. Wakefield, Mary Scharlieb, Francis Layland Barratt, Alfred Langton

**Affiliations:** Lady Mayoress; Lady Mayoress; Lady Mayoress; Chairman of Royal Free Hospital; Chairman of Royal Free Hospital; Hon. Treasurer; Chairman of Weekly Board


*To the Editor of* The Hospital.

### An Appeal for £200,000.

*To the Editor of* The Hospital.

Sir,—The fearful expenditure of life must be the first thought aroused by this devastating war, but the preciousness of life is no new thought to sensible people. After all, the wholesale destruction of fellow-creatures by this great calamity is not more appalling than the chronic ignorance and neglect which have prevailed so widely hitherto in regard to the preservation and fostering of human life almost from its source. The truth is forcibly brought home to us when we realise that, but for the want of enlightened and modernised methods in the care of maternity and childhood, *we British could have put into the field a million more men than are available to-day.*

For the past five years the Royal Free Hospital has been developing a project for further safeguarding infant life by the establishment of ante-natal and other special clinics. The result of this important work has given rise to a more extensive scheme, and one which must commend itself to everyone imbued with public spirit. It has made these specialised clinics the consultative centre of a number of departments devoted to the promotion of infant welfare at all stages.

The sum of £200,000 is now urgently needed to put this scheme into constructive and permanent shape, involving additional accommodation for maternity as well as for other patients needing special treatment, and although the present war has laid many exacting demands upon the nation, there are immediate reasons for urging the present appeal. In aid of this appeal, a generous benefactor has come forward and presented the hospital with an adjacent site in the Gray's Inn Road, upwards of an acre in extent.

Before the war, the Royal Free Hospital, in addition to enlarging and modernising its pathological department, had started a new building to provide increased facilities for out-patient work; and, though the financial position of the institution—like others of its kind—is affected by the war, it is responding nobly to its patriotic obligations. It placed this new building at the disposal of the military authorities, and therein a large number of our wounded are being nursed back to health and strength. A generous legacy enabled the hospital authorities to wipe off a debt on this building, and now the task arises for meeting new demands of national importance.

The Royal Free Hospital is a general hospital for men, women, and children patients, and allied to it is the London (R.F.H.) School of Medicine for Women. It is the only hospital in London that opens its doors exclusively to women students. The school has doubled its entry of students this term, evidence of the growing influence that the qualified woman must have in the medical profession.

When this present project for making good the special losses now being suffered by the nation has reached its full development the Royal Free Hospital will be able to afford medical education to our countrywomen on a scale which has been impossible hitherto, and is now more than ever imperative. It is proposed to commence the first block of the new building as soon as funds and circumstances allow, and we ask all interested to help in providing the necessary amount, feeling confident that they will realise the benefits to be conferred upon the future generations of our Empire.

Donations or promises should be addressed to Sir Francis Layland Barratt, Bart., M.P. (Hon. Treasurer), Royal Free Hospital, Gray's Inn Road, London, W.C., where all information concerning the appeal can be obtained from the Secretary, Special Appeal Fund.

L. E. Beaufort, Adeline M. Bedford, S. F. Wakefield (Lady Mayoress), Mary Scharlieb, Sandwich (Chairman of Royal Free Hospital), Francis Layland Barratt (Hon. Treasurer), Alfred Langton (Chairman of Weekly Board).

